# Clinical characteristics, frailty, and one-year survival outcomes in centenarian patients presenting to the emergency department

**DOI:** 10.1186/s12877-026-07593-6

**Published:** 2026-05-02

**Authors:** İskender Aksoy, Bilge Olgun Keleş, Hayriye Bektaş Aksoy

**Affiliations:** 1 Department of Emergency Medicine, Giresun University Faculty of Medicine, Giresun, Turkey; 2 Department of Anesthesiology, Giresun University Faculty of Medicine, Giresun, Türkiye; 3 Department of Chest Disease, Giresun University Faculty of Medicine, Giresun, Türkiye

**Keywords:** Centenarians, Clinical Frailty Scale, Emergency department, Frailty, Geriatric emergency medicine, Survival analysis

## Abstract

**Background:**

Centenarian patients constitute a rapidly growing yet understudied population in emergency medicine. Evidence regarding prognostic factors and one-year outcomes in individuals aged 100 years and older presenting to the emergency department remains limited.

**Methods:**

This retrospective observational study was conducted in the emergency department of Giresun Training and Research Hospital and included centenarian patients presenting between 2010 and 2023. A total of 160 emergency department visits from 83 unique patients were evaluated. Demographic characteristics, clinical variables, comorbidities, frailty indices, and laboratory parameters obtained at admission were recorded. Frailty was assessed using a modified frailty index excluding functional dependence (mFI-4) and the Clinical Frailty Scale (CFS). The primary outcome was one-year all-cause mortality. Kaplan–Meier survival analysis and Cox proportional hazards regression analysis were performed at the patient level using the index emergency department visit.

**Results:**

In the descriptive visit-level analysis, non-survivor visits showed higher hospitalization frequency and less favorable inflammatory and renal function marker profiles than survivor visits, while pulmonary diseases were more frequent among non-survivors and cardiovascular diseases were more common among survivors. Modified frailty index scores did not differ significantly between groups. Higher CFS categories were associated with shorter median survival times, although Kaplan–Meier analysis showed no statistically significant separation between frailty categories. In Cox regression analysis, hospitalization at the index emergency department visit and higher blood urea nitrogen levels remained independently associated with one-year mortality.

**Conclusion:**

In centenarian patients presenting to the emergency department, traditional comorbidity-based frailty indices show limited discriminatory value for one-year mortality. Acute clinical presentation and laboratory parameters reflecting inflammatory burden, renal function, and physiological reserve appear to be more closely associated with outcomes.

**Clinical trial registration:**

The study is not registered in a clinical trial registry.

## Introduction

The global increase in life expectancy has led to a steady rise in the number of centenarians worldwide, including in Türkiye. According to national demographic data, the population aged 100 years and older has increased markedly over the past decades, creating new challenges for healthcare systems, particularly emergency care services [1]. As this extremely old population grows, emergency departments are increasingly encountering centenarian patients, who often present with complex clinical profiles and limited physiological reserve.

Previous studies have shown that emergency department utilization among centenarians, although relatively uncommon, is frequently associated with acute and severe clinical conditions requiring hospital admission [2, 3]. Hospitalization and short-term mortality rates in this age group are reported to be high, reflecting the vulnerability of centenarian patients to acute illness [4]. However, most available data originate from Western populations, and emergency department–based studies focusing specifically on centenarians remain scarce. In Türkiye, emergency medicine data in very old populations are limited, with most studies focusing on broader elderly groups or nonagenarians rather than centenarians [5, 6].

Despite the growing clinical relevance of centenarian patients in emergency settings, there is limited evidence regarding their one-year outcomes and prognostic characteristics following emergency department admission. In particular, data integrating demographic features, reasons for admission, laboratory parameters, and survival outcomes are lacking. Therefore, the aim of this study was to evaluate the clinical characteristics, laboratory findings, and one-year survival outcomes of centenarian patients presenting to the emergency department, and to explore factors associated with mortality in this unique and underrepresented population.

## Materials and methods

### Study design and setting

This retrospective observational study was conducted at the emergency department of Giresun Training and Research Hospital, a tertiary-care center serving the Eastern Black Sea region of Türkiye. Centenarian patients who presented to the emergency department between January 1, 2010, and December 31, 2023, were evaluated. The study protocol was approved by the local ethics committee (BAEK-224, decision no: 19.02.2025/02), and the requirement for informed consent was waived due to the retrospective nature of the study.

### Study population

**Fig. 1:**
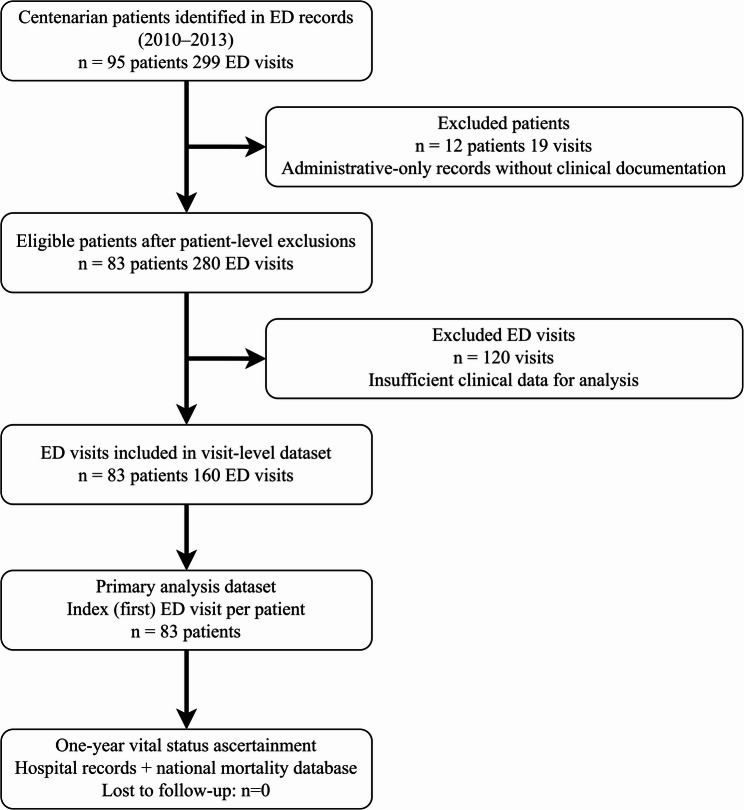
STROBE-style flow diagram of patient and visit selection. Flow diagram illustrating identification of centenarian patients, exclusion of records without analyzable clinical documentation, and the final datasets used for visit-level and patient-level analyses

All patients aged one hundred years or older who presented to the emergency department during the study period were screened for eligibility. Emergency department visits were included if patients met the age criterion and had available clinical and laboratory data. Patients presenting with cardiac arrest and those with records containing insufficient clinical documentation (e.g., administrative records without examination notes, laboratory results, or discharge summaries) were excluded. For descriptive visit-level summaries, each eligible emergency department visit was recorded as a separate clinical episode. For patient-level analyses, the first eligible visit with complete clinical data during the study period was considered the index emergency department visit. During the initial screening period, 95 centenarian patients accounting for 299 emergency department visits were identified. Among these, 12 patients (19 visits) had records containing only administrative registration data without analyzable clinical documentation (e.g., no examination notes, laboratory results, or discharge summaries) and were therefore excluded. Among the remaining 83 patients (280 visits), 120 visits lacked sufficient clinical documentation for analysis and were excluded at the visit level. Consequently, the final analytical dataset consisted of 83 patients and 160 emergency department visits with complete clinical information (Fig. 1). One-year vital status was determined using hospital records and the national mortality database; therefore, no patients were lost to follow-up. Because centenarians represent an extremely rare population, a formal a priori sample size or power calculation was not performed. Instead, all eligible centenarian emergency department visits during the predefined study period were included in the analysis.

### Data collection

Data were extracted retrospectively from hospital electronic medical records. Demographic variables included age and sex at the time of emergency department admission. Clinical variables comprised the reason for emergency department admission, hospitalization status, requirement for intensive care unit admission, and length of hospital stay. Comorbidity burden was evaluated using documented individual comorbid conditions and summarized with the Charlson Comorbidity Index. Laboratory parameters obtained at emergency department admission included hematological, biochemical, inflammatory, nutritional, and cardiac biomarkers. Vital status at one year after the index emergency department visit was determined using hospital medical records and national mortality databases.

### Frailty assessment

Frailty was assessed using the Modified Frailty Index (mFI) and the Clinical Frailty Scale (CFS). The original mFI-5 consists of five components: diabetes mellitus, chronic obstructive pulmonary disease, congestive heart failure, hypertension requiring medication, and functional dependence. Due to the retrospective design of the study and the limited granularity of functional status documentation in centenarian patients, the functional dependence component could not be reliably classified. Therefore, frailty was assessed using a modified mFI-4, calculated by excluding the functional dependence component. The remaining four comorbidity-based components were summed to generate an mFI-4 score ranging from 0 to 4. This approach was adopted to minimize misclassification bias in this extremely old population. In addition, global clinical frailty was evaluated using the Clinical Frailty Scale, scored from 1 (very fit) to 9 (terminally ill), based on documented clinical and functional information available at presentation.

### Laboratory measurements

Laboratory measurements obtained at emergency department admission included complete blood count, renal and liver function tests, electrolytes, inflammatory markers, nutritional markers, and cardiac biomarkers. Inflammatory and nutritional indices were calculated using standard formulas, including the neutrophil-to-lymphocyte ratio (NLR), platelet-to-lymphocyte ratio (PLR), lymphocyte-to-monocyte ratio (LMR), systemic immune-inflammation index (SII).

The primary outcome of the study was one-year all-cause mortality, defined as death from any cause within one year after the index emergency department visit. Vital status was determined using hospital records and the national mortality database.

### Outcome measures

The primary outcome of the study was one-year all-cause mortality, defined as death from any cause within one year following the index emergency department visit. Only emergency department visits with complete clinical documentation were eligible for inclusion in the analysis. For patient-level analyses, the first eligible visit with complete clinical data during the study period was considered the index emergency department visit.

### Statistical analysis

All statistical analyses were performed using IBM SPSS V 22.0 (IBM Corp., Armonk, NY, USA). The normality of continuous variables was assessed using the Kolmogorov–Smirnov test together with graphical inspection of histograms and Q–Q plots. Variables showing normal distribution were analyzed using the independent samples t test, whereas variables not normally distributed were compared using the Mann–Whitney U test. Categorical variables were compared using the chi-square test, and the Fisher’s exact test was applied when expected cell counts were less than five. Bonferroni correction was applied for multiple comparisons in subgroup analyses. The adjusted significance threshold was calculated by dividing the conventional alpha level (0.05) by the number of comparisons performed. Survival analysis was performed using the Kaplan–Meier method to estimate 1-year survival according to modified frailty index (mFI-4) and Clinical Frailty Scale categories. Differences between survival curves were evaluated using the log-rank (Mantel–Cox) test. Cox proportional hazards regression analysis was performed at the patient level using the index emergency department visit to identify predictors of one-year mortality. Variables that were statistically significant in the univariable analysis were considered for inclusion in the multivariable model. Because blood urea nitrogen and serum creatinine both reflect renal function and may convey overlapping information, only blood urea nitrogen, which showed the stronger association in the univariable analysis, was retained in the final multivariable model to avoid redundancy. Hazard ratios were reported with 95% confidence intervals. Continuous variables were presented as median (IQR), and categorical variables were expressed as n (%). A p value < 0.05 was considered statistically significant.

## Results

A total of 160 emergency department visits by eighty-three centenarian patients were included in the analysis. Among the 83 patients, 48 (57.8%) had a single ED visit, 11 (13.3%) had two visits, 10 (12.0%) had three visits, and 14 (16.9%) had four or more visits during the study period. Baseline demographic and frailty characteristics were evaluated using the index (first) emergency department visit for each patient (Table 1). Age and sex distribution were similar between patients who survived and those who died within one year (*p* = 0.159 and *p* = 0.189, respectively). Similarly, the modified frailty index calculated using mFI-4, Charlson Comorbidity Index, and Clinical Frailty Scale scores did not differ significantly between the two groups.

**Table 1:** Baseline characteristics of centenarian patients according to one-year survival status (patient-level analysis, index ED visit, *n* = 83)

	Non-survivor		Survivor		
	*n*	Median (IQR) or *n* (%)	*n*	Median (IQR) or *n* (%)	*p*
Age, years	48	101 (100–101.8)	35	101 (100–103)	0.159
Gender					
Female	43	89.6	34	97.1	0.189
Male	5	10.4	1	2.9	
mFI-4 score	48	1 (1–2)	35	1 (1–1)	0.120
Diabetes Mellitus	6	12.5	2	5.7	0.301
COPD	9	18.8	7	20.0	0.887
Congestive Heart Failure	13	27.1	4	11.4	0.081
Hypertension	35	72.9	21	60.0	0.480
Charlson Comorbidity Index	48	2 (1–3)	35	2 (1–3)	0.907
Clinical Frailty Scale (1–9)	48	7 (7–8)	35	7 (6–8)	0.176

Baseline characteristics were calculated using the index (first) emergency department visit for each patient. *mFI-4* Modified Frailty Index-4, *COPD* Chronic Obstructive Pulmonary Disease. Continuous variables were compared using the Mann–Whitney U test. Categorical variables were compared using the Pearson chi-square test or Fisher’s exact test, as appropriate

Visit-level clinical characteristics across all eligible emergency department visits are presented descriptively in Table 2. In this secondary visit-level analysis, non-survivor visits showed higher white blood cell count, neutrophil count, blood urea nitrogen, NLR, and SII values than survivor visits. Hospitalization was more frequent among non-survivor visits, whereas intensive care unit admission and length of stay appeared broadly similar between groups. Pulmonary diseases were observed more commonly among non-survivor visits, while cardiovascular diseases were more frequent among survivor visits. Because some patients contributed multiple visits, these visit-level findings are presented descriptively and should be interpreted with caution.

**Table 2:** Visit-level laboratory parameters and emergency department characteristics according to one-year survival status (secondary analysis of 160 ED visits from 83 patients)

	Non-survivor		Survivor	
	*n*	Median (IQR) or *n* (%)	*n*	Median (IQR) or *n* (%)
Laboratory parameters				
White blood cell count (×10⁹/L)	102	8.80 (6.71–12.52)	58	7.73 (6.20–9.26)
Neutrophil count (×10⁹/L)	102	6.69 (4.67–9.77)	58	5.47 (3.97–6.82)
Lymphocyte count (×10⁹/L)	102	1.39 (0.92–1.79)	58	1.50 (1.07–2.24)
Monocyte count (×10⁹/L)	102	0.54 (0.37–0.85)	58	0.50 (0.34–0.65)
Platelet count (×10⁹/L)	102	217 (177–283)	58	227 (184–274)
Hemoglobin (g/dL)	102	12.0 (10.7–13.1)	58	11.7 (10.4–12.4)
Hematocrit (%)	102	36.5 (32.6–40.4)	58	37.0 (35.0–38.7)
Serum creatinine (mg/dL)	98	0.88 (0.69–1.61)	54	0.94 (0.78–1.19)
Blood urea nitrogen (mg/dL)	98	60 (44–97)	54	47 (38–62)
eGFR (mL/min/1.73 m²)	98	64.7 (31.2–83.6)	54	57.4 (43.5–72.4)
ALT (U/L)	100	11 (8–19)	52	12 (8–16)
AST (U/L)	100	21 (16–31)	52	22 (16–26)
Total bilirubin (mg/dL)	93	0.60 (0.36–0.87)	47	0.49 (0.32–0.76)
Direct bilirubin (mg/dL)	93	0.19 (0.12–0.35)	47	0.15 (0.09–0.22)
Sodium (mmol/L)	97	139 (135–143)	51	139 (138–141)
Chloride (mmol/L)	97	102 (97–107)	51	104 (100–105)
Potassium (mmol/L)	97	4.5 (4.0–4.8)	51	4.3 (3.8–4.5)
Inflammatory indices				
NLR	102	4.74 (2.98–9.15)	57	3.20 (1.86–5.59)
PLR	102	167.51 (107.68–249.41)	58	145.38 (109.77–221.58)
LMR	102	2.58 (1.61–3.98)	58	3.33 (1.95–4.98)
SII	102	1071 (634–1881)	58	756 (397–1357)
Emergency department characteristics				
Hospitalization status				
Yes	60	58.8	20	34.5
No	42	41.2	38	65.5
ICU admission				
Yes	29	48.3	9	42.9
No	31	51.7	12	57.1
Length of stay (days)	59	8.0 (4.0–15.0)	21	5.0 (3.5–9.5)
Reason for admission				
Pulmonary diseases	26	25.5	10	17.2
Neurological diseases	19	18.6	13	22.4
Gastroenterology-related disease	20	19.6	10	17.2
Cardiovascular diseases	7	6.9	16	27.6
Infectious diseases	11	10.8	4	6.9
Renal disease	11	10.8	3	5.2
Trauma	4	3.9	2	3.4
Electrolyte disorders	4	3.9	0	0

This table summarizes visit-level measurements across all eligible emergency department visits (160 visits from 83 patients)

*eGFR* Estimated Glomerular Filtration Rate, *ALT* Alanine Aminotransferase, *AST* Aspartate Aminotransferase, *NLR* Neutrophil-to-Lymphocyte Ratio, *PLR* Platelet-to-Lymphocyte Ratio, *LMR* Lymphocyte-to-Monocyte Ratio, *SII* Systemic Immune-Inflammation Index (Neutrophils × Platelets / Lymphocytes)

**Fig. 2:**
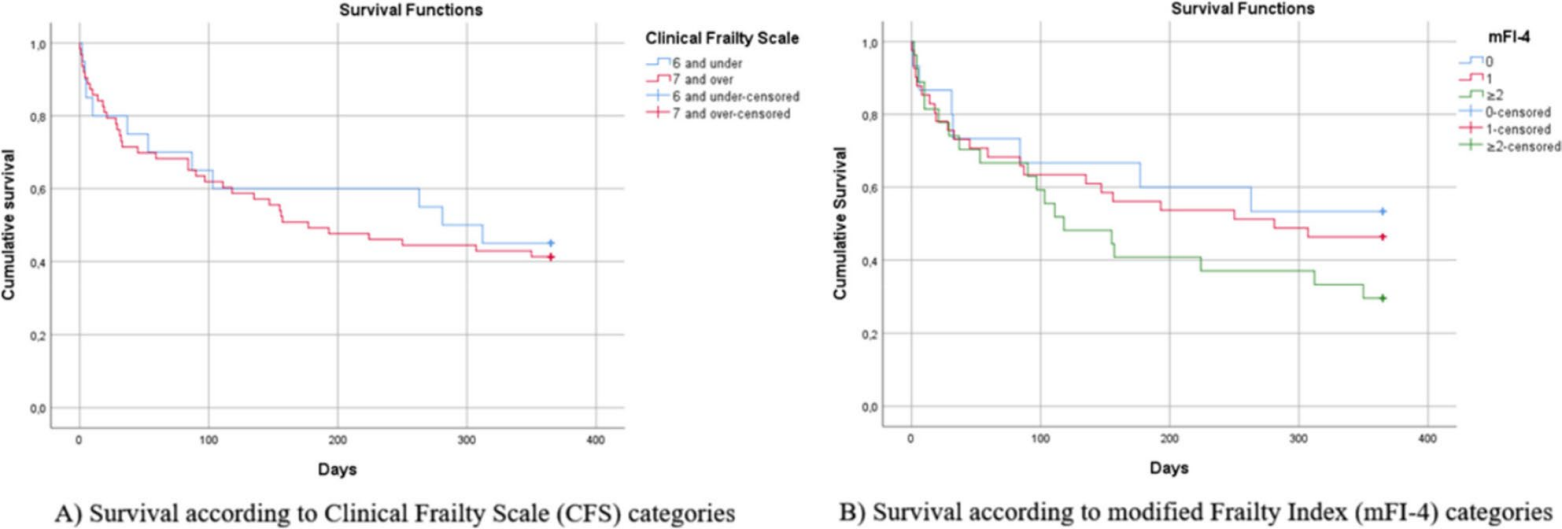
Kaplan–Meier survival curves for one-year mortality in centenarian patients (Suvival according to Clinical Frailty Scale (**A**) and modified Frailty Index (**B**)

Kaplan–Meier survival analysis performed at the patient level using the index emergency department visit demonstrated no significant difference in one-year survival across mFI-4 categories (log-rank [Mantel–Cox] test, *p* = 0.350) or Clinical Frailty Scale categories (*p* = 0.723). Although median survival time tended to decrease with increasing frailty score, these differences did not reach statistical significance (Table 3). The Kaplan–Meier survival curves are shown in Fig. 2A and B.

**Table 3:** Kaplan–Meier survival analysis according to modified frailty index (mFI-4) and Clinical Frailty Scale (patient-level analysis, *n* = 83)

mFI-4 score	Non-survivor, *n* (%)	Survivor, *n* (%)	Median survival time (days)	95% CI
0	7 (14.6)	8 (22.9)	234	156–311
1	22 (45.8)	19 (54.3)	215	167–263
≥ 2	19 (39.6)	8 (22.9)	178	122–234
Overall survival			206	173–240
Clinical Frailty Scale				
6 and under	11 (22.9)	9 (25.7)	281	174–388
7 and over	37 (77.1)	26 (74.3)	177	63–291
Overall survival			224	73–375

Survival analysis was performed at the patient level using the index (first) emergency department visit for each patient. Frailty was assessed using the modified frailty index (mFI-4), calculated by excluding the functional dependence component from the mFI-5 due to limited granularity of functional status data in retrospective records. Survival differences between groups were evaluated using the log-rank (Mantel–Cox) test. The log-rank p value was 0.350 for mFI-4 categories and 0.723 for Clinical Frailty Scale categories

Cox proportional hazards regression analysis was performed to identify independent predictors of one-year mortality. In univariable analysis, hospitalization at the index emergency department visit, higher neutrophil-to-lymphocyte ratio, elevated blood urea nitrogen, and higher serum creatinine levels were associated with increased mortality risk. In the multivariable model, hospitalization at the index emergency department visit (HR 1.92, 95% CI 1.02–3.61, *p* = 0.043) and blood urea nitrogen (HR 1.013, 95% CI 1.008–1.019, *p* < 0.001) remained independently associated with one-year mortality, whereas the association with NLR did not retain statistical significance (Table 4). Although serum creatinine was statistically significant in the univariable analysis, it was not included in the final multivariable model because both serum creatinine and blood urea nitrogen reflect renal function, and blood urea nitrogen showed the stronger univariable association.

**Table 4:** Cox proportional hazards regression analysis for predictors of one-year mortality in centenarian patients

	Univariable HR(95% CI)	*p*	Multivariable HR(95% CI)	*p*
Male sex	2.340 (0.924–5.924)	0.073		
Hospitalization	2.112 (1.165–3.827)	0.014	1.920 (1.022–3.608)	0.043
NLR	1.028 (1.000–1.056)	0.047	1.021 (0.989–1.055)	0.201
PLR	1.001 (1.000–1.002)	0.112		
LMR	0.981 (0.921–1.045)	0.547		
SII	1.000 (1.000–1.000)	0.578		
Blood urea nitrogen (mg/dL)	1.014 (1.009–1.020)	< 0.001	1.013 (1.008–1.019)	< 0.001
Serum creatinine (mg/dL)	1.671 (1.283–2.176)	< 0.001		
eGFR (mL/min/1.73 m²)	0.999 (0.992–1.007)	0.876		
Charlson Comorbidity Index	0.965 (0.804–1.157)	0.697		
Clinical Frailty Scale (1–9)	1.137 (0.911–1.419)	0.258		
mFI-4 score	1.229 (0.891–1.696)	0.210		

Cox regression analysis was performed at the patient level using the index (first) emergency department visit

*HR* Hazard ratio, *CI* Confidence interval, *NLR* Neutrophil-to-lymphocyte ratio, *PLR* Platelet-to-lymphocyte ratio, *LMR* Lymphocyte-to-monocyte ratio, *SII* Systemic immune-inflammation index, *eGFR* Estimated glomerular filtration rate, *mFI-4* Modified Frailty Index-4

## Discussion

This study evaluated clinical and laboratory predictors of one-year mortality among centenarian patients presenting to the emergency department. The main findings of the study can be summarized as follows. First, traditional frailty and comorbidity indices, including the Clinical Frailty Scale and modified Frailty Index, showed limited discriminatory ability for predicting one-year mortality in this exceptionally old population. Second, acute clinical characteristics observed at the index emergency department visit, particularly hospitalization status, were associated with increased mortality risk. Third, laboratory markers reflecting acute physiological stress, especially blood urea nitrogen levels, were identified as independent predictors of one-year mortality in the multivariable Cox regression analysis. Together, these findings suggest that acute clinical severity and physiological disturbances may provide more meaningful prognostic information than conventional frailty measures in centenarian patients.

In this single-center study focusing on centenarian patients presenting to the emergency department, several important clinical characteristics were identified. Previous studies have demonstrated that emergency department utilization among individuals aged 100 years and older, although relatively uncommon, has been increasing in parallel with population aging and is frequently associated with acute, high-severity clinical conditions rather than low-acuity complaints [2, 3, 5, 7]. In line with these observations, our cohort exhibited a high rate of hospital admission, particularly among patients who died within one year, supporting the notion that emergency department presentation in centenarians often reflects clinically significant acute illness. Similar findings have been reported in previous studies, where emergency department visits among centenarians frequently result in hospitalization and are associated with short- and mid-term mortality [4, 8–11]. Notably, pulmonary conditions were more prevalent among non-survivors in our study, whereas cardiovascular diseases were more frequently observed among survivors. This finding is consistent with previous reports suggesting that acute respiratory illnesses may be associated with a higher mortality risk in extremely old individuals compared with chronic cardiovascular presentations, which often represent more stable disease states at the time of emergency department admission [2, 7].

In the present study, hospitalization following emergency department admission was more frequent among centenarian patients who died within one year, whereas intensive care unit use and length of hospital stay appeared broadly similar between survivors and non-survivors. Previous studies have consistently reported high hospitalization rates among centenarians presenting to the emergency department, reflecting the severity of acute illness in this population [4, 8, 9]. At the same time, the prognostic implications of hospitalization remain heterogeneous, as several studies have associated hospital admission with increased short- to mid-term mortality, whereas others have reported that aggressive inpatient or intensive care management does not necessarily improve one-year survival in centenarians [10–13]. Our findings are consistent with this body of evidence, suggesting that hospitalization in centenarian patients may primarily serve as a marker of acute clinical severity rather than an independent determinant of survival. The absence of significant differences in intensive care unit utilization and hospital length of stay between survivors and non-survivors further supports the concept that outcomes in this age group may be driven more by the underlying acute physiological insult than by the intensity or duration of inpatient care.

Frailty has been widely recognized as an important determinant of adverse outcomes in older adults; however, its assessment in centenarians remains methodologically challenging. The modified frailty index (mFI-5), which incorporates functional dependence as one of its core components, has been extensively used in surgical and medical cohorts and has demonstrated prognostic value in younger elderly populations [14]. Nevertheless, evidence regarding the performance of mFI-based frailty assessment in centenarians is limited, and recent reviews have highlighted substantial heterogeneity in frailty definitions and outcome associations in very old populations [13]. In studies including extremely old individuals, frailty scores often cluster at higher levels, reducing their discriminative capacity for mortality and other one-year outcomes [4, 8]. In centenarian populations, frailty scores tend to cluster within a relatively narrow high-risk range, which may reduce their ability to differentiate mortality risk between individuals. As a result, acute clinical conditions and physiological disturbances observed at emergency department presentation may provide more informative prognostic signals than traditional frailty indices in this age group. In addition, the multivariable Cox regression analysis identified hospitalization at the index emergency department visit and blood urea nitrogen levels as independent predictors of one-year mortality. These findings suggest that acute clinical severity and markers of physiological stress may provide stronger prognostic signals than traditional frailty or comorbidity indices in centenarian patients.

An additional challenge relates to the assessment of functional dependence, particularly in retrospective emergency department–based studies. Functional status encompasses a broad spectrum of abilities, ranging from minor limitations in activities of daily living to complete dependency, yet these differences are often simplified when functional dependence is treated as a binary variable. Previous studies that applied mFI-5 in very old populations often relied on simplified or proxy definitions of functional dependence, potentially introducing subjectivity and misclassification [14]. In the present study, frailty was therefore assessed using a modified mFI-4, excluding the functional dependence component, to improve objectivity and reproducibility. Despite this adjustment, frailty as measured by mFI-4 was not significantly associated with one-year mortality, supporting prior observations that comorbidity-based frailty indices may have limited prognostic value in centenarians, where acute physiological stressors and short-term illness severity appear to play a more prominent role in determining outcomes [2, 3].

In the present study, several laboratory and inflammatory parameters obtained at emergency department admission showed unfavorable patterns among non-survivor visits. In particular, renal function and inflammation-related markers such as blood urea nitrogen, neutrophil-to-lymphocyte ratio, and systemic immune-inflammation index were higher in non-survivor visits. Previous studies have emphasized that, in extremely old individuals, laboratory abnormalities at acute presentation often reflect diminished physiological reserve and systemic stress rather than isolated organ dysfunction [9, 15]. Similar findings have been reported in cohorts of very old emergency department and critically ill patients, where inflammatory and metabolic disturbances were associated with short- and mid-term outcomes, highlighting the prognostic relevance of acute physiological derangement in this population [11, 13, 16].

These laboratory findings may provide complementary prognostic information beyond traditional comorbidity-based frailty indices. In centenarian populations, where chronic disease burden and functional impairment are highly prevalent, markers reflecting acute inflammatory response and physiological stress may better capture the immediate biological impact of illness. Consistent with this interpretation, the findings of the present study suggest that laboratory parameters obtained at emergency department admission may offer clinically relevant prognostic information by reflecting the severity of acute illness superimposed on extreme biological aging [11, 16].

In the present study, Kaplan–Meier survival analysis did not demonstrate a statistically significant difference in one-year survival across modified frailty index (mFI-4) categories among centenarian patients. Although higher mFI-4 categories were associated with shorter median survival times, this trend did not reach statistical significance. These findings suggest that, in populations characterized by extreme biological aging, conventional frailty stratification may have limited discriminatory value for one-year survival. Previous studies evaluating survival patterns in centenarians have similarly reported relatively homogeneous mortality trajectories, regardless of baseline comorbidity burden or frailty classification [4, 8, 15].

Several population-based and hospital-based studies have further demonstrated that survival curves in centenarian cohorts tend to converge over time, reflecting the dominant influence of extreme age itself on one-year outcomes [13, 17, 18]. Taken together, these findings support the notion that, in very advanced age groups, survival analysis may be more informative when interpreted descriptively rather than as a tool for risk stratification based on traditional clinical indices. Although higher frailty scores were associated with shorter median survival times, neither mFI-4 nor CFS demonstrated statistically significant separation in Kaplan–Meier survival analysis, underscoring the limited discriminatory capacity of frailty measures in centenarians.

Although chronological age is widely recognized as a key determinant of prognosis in older adults, its interpretation becomes more complex in centenarian populations. The age range within this group is inherently narrow, typically spanning only a few years, which may limit the clinical impact of small statistical differences. Therefore, while age remains an important biological marker of survival, its prognostic discrimination may be reduced among individuals who have already reached extreme longevity. In such populations, acute clinical conditions, functional status, and physiological reserve may play a more prominent role in determining outcomes than chronological age alone.

The findings of the present study have several important clinical implications for the management of centenarian patients presenting to the emergency department. First, conventional frailty indices and comorbidity-based risk stratification tools may have limited utility in predicting one-year outcomes in this extremely old population. Instead, acute clinical presentation and laboratory parameters reflecting inflammatory burden, renal function, and physiological reserve appear to provide more relevant information regarding prognosis.

Several limitations of this study should be acknowledged. First, the retrospective single-center design limits the generalizability of the findings to other settings and healthcare systems. Additionally, in a small number of historical records, only administrative entries were available in the hospital information system (e.g., patient registration without accompanying clinical documentation such as examination notes, laboratory results, or discharge summaries). Because these records did not contain analyzable clinical information, they were excluded from the study. Second, although multiple emergency department visits were included in the analysis, patient-level clustering could not be fully accounted for, which may have influenced the observed associations. Third, functional status could not be assessed in a standardized and granular manner due to the retrospective nature of the data, necessitating the use of a modified frailty index excluding functional dependence. Fourth, the relatively small sample size inherent to centenarian cohorts may have limited statistical power, particularly for subgroup and survival analyses. Fifth, laboratory parameters were assessed only at emergency department admission, and longitudinal changes over time could not be evaluated. Finally, the study period overlapped with the COVID-19 pandemic, which may have influenced pulmonary presentations and mortality patterns in this very elderly population. Because specific COVID-19 status was not systematically available for all visits, the potential impact of the pandemic should be considered when interpreting the observed associations. Despite these limitations, this study provides valuable real-world data on a rarely studied population and contributes to a more nuanced understanding of prognostic factors in centenarian patients presenting to the emergency department.

## Conclusion

This study provides a comprehensive evaluation of centenarian patients presenting to the emergency department and highlights the unique prognostic characteristics of this extremely old population. Our findings indicate that traditional frailty and comorbidity-based indices have limited discriminatory value for one-year mortality in centenarians. Instead, acute clinical presentation and laboratory parameters reflecting inflammatory burden, renal function, and physiological reserve appear to provide more relevant information regarding prognosis.

These results underscore the importance of focusing on acute illness severity rather than chronic health scores when assessing centenarian patients in the emergency department. In this context, emergency department presentation may be viewed as a critical physiological stress test in centenarians, providing valuable insight into short- and mid-term prognosis. Future multicenter studies with larger cohorts are warranted to further clarify prognostic markers and optimize clinical decision-making in this rapidly growing but underrepresented population.

## Data Availability

The datasets used and/or analyzed during the current study are available from the corresponding author on reasonable request.

## References

[1] TurkStat. Elderly Statistics. Türkiye İstatistik Kurumu. 2022.

[2] Carey MR, Howell EM, McHugh MC. Emergency department use by centenarians: the 2008 Nationwide Emergency Department Sample. Prev Chronic Dis. 2013;10:E198. 10.5888/pcd10.120006 PMC3843535 24286272

[3] Mane G, Alkhouri H, Dinh M, McCarthy S. One hundred and counting: Centenarian use of emergency departments in New South Wales. Emerg Med Australas. 2019;31(4):626–31. 30866166 10.1111/1742-6723.13259

[4] Mandawat A, Mandawat A, Mandawat MK, Tinetti ME. Hospitalization rates and in-hospital mortality among centenarians. Arch Intern Med. 2012;172(15):1179–80. 22710863 10.1001/archinternmed.2012.2155 PMC4241633

[5] Bilir O, Yazici MM, Atas I, Tasci F, Ersunan G. Brain computed tomography in centenarians presenting to the emergency department: is age an indication to CT scan? Eur Rev Med Pharmacol Sci. 2023;27(15):6939–46. 37606104 10.26355/eurrev_202308_33266

[6] Senguldur E, Selki K. Today’s Problem Tomorrow’s Crisis: A Retrospective, Single-Centre Observational Study of Nonagenarians in the Emergency Department. Cureus. 2024;16(11):e73460. 39669848 10.7759/cureus.73460 PMC11634557

[7] Romano N, Fischetti A, Siri G, Castaldi A. Head CT in centenarians: emergency and non-emergency findings. Aging Clin Exp Res. 2022;34(1):201–8. 33934276 10.1007/s40520-021-01862-7

[8] Dotchin CL, Gray WK, Gaskin E, Hartley S, Walker RW. Frequency, nature and outcomes of hospital admissions in centenarians in an area of North-East England. Geriatr Gerontol Int. 2016;16(8):969–75. 26311143 10.1111/ggi.12586

[9] Saez-Nieto C, Ly-Yang F, Perez-Rodriguez P, Alarcon T, Lopez-Arrieta J, Gonzalez-Montalvo JI. Impact of hospital admission on centenarians admitted due to an acute illness. A description of a series of 165 cases and comparison with the literature. Rev Esp Geriatr Gerontol. 2019;54(6):315–20. 31301820 10.1016/j.regg.2019.04.005

[10] Pineiro-Fernandez JC, Rabunal-Rey R, Romay-Lema E, Chantres-Legaspi Y, Santos-Martinez AM, Besteiro-Balado Y et al. Trends in hospital admissions and clinical complexity in centenarians: a nationwide population-based study in Spain (2004–2020). Eur Geriatr Med. 2025;17:797–809. 10.1007/s41999-025-01362-1 PMC13109149 41288906

[11] Yu HJ, Lai ET, Yu R, Woo J. Trends and characteristics of hospitalisation and mortality among centenarians in old-age homes and communities: a territory-wide study in Hong Kong, 2012–2021. BMJ Public Health. 2025;3(2):e002879. 41262777 10.1136/bmjph-2025-002879 PMC12625965

[12] Dekker AP, Saxena PA, Westwood E, Kalla N, Sims N, Wilson P, et al. Outcomes for centenarian patients admitted with orthopaedic trauma. Surgeon. 2024;22(6):354–7. 39368884 10.1016/j.surge.2024.09.010

[13] Suh JM, Raykateeraroj N, Waldman B, Kitisin N, Haywood C, Bellomo R, et al. Characteristics, outcomes, and complications among nonagenarian and centenarian patients admitted to the intensive care unit: a scoping review. Crit Care. 2025;29(1):112. 40083001 10.1186/s13054-025-05349-z PMC11907827

[14] Weaver DJ, Malik AT, Jain N, Yu E, Kim J, Khan SN. The Modified 5-Item Frailty Index: A Concise and Useful Tool for Assessing the Impact of Frailty on Postoperative Morbidity Following Elective Posterior Lumbar Fusions. World Neurosurg. 2019;124:e626–32. 30639495 10.1016/j.wneu.2018.12.168

[15] Koyama T, Higashionna T, Maruo A, Ushio S, Zamami Y, Harada K, et al. Trends in places and causes of death among centenarians in Japan from 2006 to 2016. Geriatr Gerontol Int. 2022;22(8):675–80. 35739616 10.1111/ggi.14416

[16] Solakoglu GA, Nuhoglu C, Al B, Adak NA, Arslan B. Prognostic factors influencing survival in nonagenarian patients admitted to the emergency department: a retrospective study. BMC Geriatr. 2025;25(1):391. 40448058 10.1186/s12877-025-06047-9 PMC12123847

[17] Evans CJ, Ho Y, Daveson BA, Hall S, Higginson IJ, Gao W, et al. Place and cause of death in centenarians: a population-based observational study in England, 2001 to 2010. PLoS Med. 2014;11(6):e1001653. 24892645 10.1371/journal.pmed.1001653 PMC4043499

[18] Mao F, Zhang W, Yin P, Wang L, You J, Liu J, et al. Epidemiological characteristics of centenarian deaths in China during 2013–2020: A trend and subnational analysis. Chin Med J (Engl). 2024;137(13):1544–52. 37718285 10.1097/CM9.0000000000002823 PMC11230835

